# Oral Leiomyoma in an Adult Male: A Case Report

**DOI:** 10.2174/1874210601711010520

**Published:** 2017-10-24

**Authors:** Amanda Phoon Nguyen, Agnieszka M Frydrych

**Affiliations:** School of Dentistry, University of Western Australia, Crawley, Western Australia

**Keywords:** Oral Leiomyoma, Hard Palate, Benign neoplasms, Histopathological assessment, Eosinophilic cytoplasm, Interlacing fascicles

## Abstract

**Introduction:**

Oral leiomyomas are rare, benign neoplasms of smooth muscle origin, presenting as a solitary, asymptomatic, nodular mass.

**Case Presentation:**

Here we present the case of a 54-year-old male with a lesion in the midline of his hard palate, which was diagnosed as a localized benign leiomyoma, along with a review of the recent literature.

**Discussion:**

Diagnosis of a leiomyoma must be based on the histopathological assessment of tissue as the clinical appearance is non-specific. The peak prevalence of head and neck leiomyoma is observed in the 4^th^ and 5^th^ decade of life with uncertain gender predilection. Histological features include interlacing fascicles of smooth muscle small cells with eosinophilic cytoplasm. Complete excision is usually curative and recurrence is rare.

**Conclusion:**

Due to their rare nature, it is important that cases of oral leiomyoma can be reported in the literature to improve our understanding of this entity.

## 1 INTRODUCTION

A leiomyoma is a circumscribed, benign tumor composed of intersecting bundles of mature smooth muscle cells, occurring most frequently in the uterine myometrium, gastrointestinal tract, skin and the lower extremities [1]. The most common sites for leiomyoma are in the uterus, with about 95% involving the female genital tract, followed by skin [2-4]. As the oral cavity is bereft of smooth muscles except in blood vessel wall, this tumor is rare in the head and neck region with reported incidence of 0.065 to 1% [4, 5], accounting for 0.42% of all soft-tissue neoplasms in the oral cavity [5]. A review of the literature by Leung and Wong in 1990 yielded only 124 cases reported from 1884 to 1987 [6]. Oral leiomyomas can appear at any age, however its peak age of incidence is between 40 and 49 years of age, with over 65 percent being found in patients older than 30 years of age [3]. Currently, the presence of any gender-specific predilection is not clear [2, 3, 7].

As smooth muscle is scarce in the oral cavity, it has been hypothesized that leiomyomas may originate from the tunica media, or the smooth muscle of excretory ducts of salivary glands [2]. Glass *et al*. have proposed that tongue leiomyomas may originate from the ductus lingualis and circumvallate papilla [8]. The most commonly affected intraoral sites involve the posterior portion of the tongue, followed by palate, buccal mucosa, lips and salivary glands which together account for over 80% of cases [3]. Other less frequent locations are the floor of the mouth and the gingiva [3]

Leiomyomas are generally asymptomatic, although tumors can occasionally become painful. The diagnosis is mainly determined by histological studies due to their non-specific clinical appearance [2]. Typically, a solid leiomyoma presents as a slowly enlarging, asymptomatic, firm, submucosal mass, with a greyish or bluish tone [9]. The colour of the lesion may also be similar to the adjacent mucosa [3]. The lesion is smooth and rarely ulcerates [9]. The size of the lesion may range from a few millimetres to several centimetres [3].

The World Health Organization (WHO) classifies leiomyomas into three histological types: solid leiomyoma (25%), vascular leiomyoma, also known as angioleiomyoma or angiomyoma (74%) and leioblastoma, also known as leiomyoblastoma or ephitheloid leiomyoma (1%) [3, 10, 11]. According to Damm and Neville, a solid leiomyoma is histologically very different from angioleiomyoma, and the two entities should therefore be regarded as separate tumors [12].

We hereby present a case of an oral leiomyoma involving the hard palate and a literature review.

## 2 CASE PRESENTATION

A 54-year-old male patient presented with the chief complaint of an asymptomatic swelling in the midline of his hard palate, which he first noted about 2 months prior. The patient was not aware of any other oral mucosal problems. His medical history was essentially non-contributory. He suffered from allergic rhinitis and was taking systemic antihistamines and using a mometasone furoate nasal spray. He was a non-smoker but reported a brief past smoking history.

Intraoral examination revealed the presence of a discrete, raised, mid-palatal ovoid lesion with a bluish appearance, measuring 6mm by 4mm. Subtle reticular, erythematous lesions were also noted to affect the gingiva and the left buccal mucosa. These reticular, erythematous lesions involving his gingiva are shown in (Fig. **1a**). No other abnormalities were identified. Extraoral examination was unremarkable.

An excisional biopsy was performed of the palatal swelling and a tissue sample measuring 12mm x 7mm x 4mm was submitted for routine histopathology. Fig. (**1b**) shows a post-operative photograph illustrating the site and extent of the excision.

The histopathology report indicated that the lesion was a leiomyoma. Immunochemistry for S100 was negative and smooth muscle actin was strongly positive. Incisional biopsy of the left maxillary buccal gingiva indicated the presence of oral lichen planus (WHO diagnostic criteria). In view of the mucosal lesions, the patient was also referred for a series of routine blood tests to ensure the absence of haematinic deficiencies. Fasting glucose levels and the presence of anti-skin antibodies was also ascertained. All results returned within normal limits.

### 2.1 Histopathological Features

A well-circumscribed, encapsulated mass was visible within the subepithelial collagen, as seen in Fig. (**2**). This was composed of interlacing fascicles of smooth muscle with small angulated vasculature lined by a simple endothelium. No evidence of malignancy was seen. (Table **1**) summarizes the histopathological features of a solid leiomyoma.

### 2.2 Immunohistochemistry

Immunohistochemically, leiomyomas are reactive with vimentin, desmin, α-smooth muscle actin (SMA), and muscle specific actin [13]. Desmin, actin, H-caldesmon and vimentin are diffusely and strongly positive in the neoplastic cells [3] Most tumor cells are also positive for smooth muscle actin and Myoglobin. The Ki-67 index is usually >5% [3]. The immunohistochemical technique is an important aid in diagnosis, in which these tumors usually express immunoreactivity for SMA and negativity for the S-100 protein. In this case, as seen in Figs. (**3a** and **3b**), immunochemistry for S100 was negative and smooth muscle actin was strongly positive.

The diagnosis reached from his left gingival biopsy was oral lichen planus (OLP). Direct immunofluorescence was performed and revealed no diagnostic features.

## 3 DISCUSSION

To the best of our knowledge, this is the first case of concurrent OLP and leiomyoma in the same patient. Given that OLP is a common oral mucosal disease, the association is likely a coincidental one.

The diagnosis of a leiomyoma must be based on the histopathological assessment of tissue as leiomyomas cannot be differentiated from other mesenchymal tumours on clinical presentation alone. The differential diagnoses of an oral leiomyoma are summarized in (Table **2**).

Table (**3**) summarizes recent cases of oral leiomyoma as described in the available literature.

In addition to routine histopathology, immunochemistry may be an invaluable tool in ensuring accurate diagnosis. For example, a myofibroma can be distinguished by the central zonal phenomenon with vimentin and SMA stains, while a hemangiopericytoma does not present with lesional cells arranged in long bundles or fascicles, and is negative for smooth muscle-specific antibodies [11]. Neural tumors usually stain positively for S-100 protein and neuron-specific enolase but not for desmin and smooth muscle actin [11].

Of particular importance is differentiating leiomyoma from its malignant counterpart, a leiomyosarcoma, especially a low-grade leiomyosarcoma. The presence of mucosal ulceration should raise suspicion of malignancy. Histopathologically, the presence of mitoses is concerning. Lesions with 5 or more mitotic figures per 10 high-power fields should be considered to have malignant behavior, while fewer than two mitotic figures per 10 high-magnification fields are is indicative of a good prognosis (Gonzalez Sanchez) [11]. Molecular markers such as PCNA, bcl-2, CDK4, p53 and MDM2, may be useful in differentiating benign from malignant tumours [11].

Treatment of leiomyoma is surgical and excision is considered curative. There is consensus across the literature that recurrences are extremely infrequent. The patient was reviewed 1 year post excision with no recurrence.

## CONCLUSION

Oral leiomyomas are rare, benign smooth muscle neoplasms. Intra-orally, they tend to occur on the posterior portion of the tongue, palate, buccal mucosa, lips and salivary glands. Because of their relative rarity and non-specific clinical appearance, diagnosis may be a challenge. Diagnosis requires histopathological assessment, and the use of stains such as smooth muscle actin may be invaluable. It is important that cases of oral leiomyoma are reported in the literature to improve our understanding of this entity. Treatment is surgical and excision is associated with a very low recurrence rate.

## Figures and Tables

**Fig. (1a):**
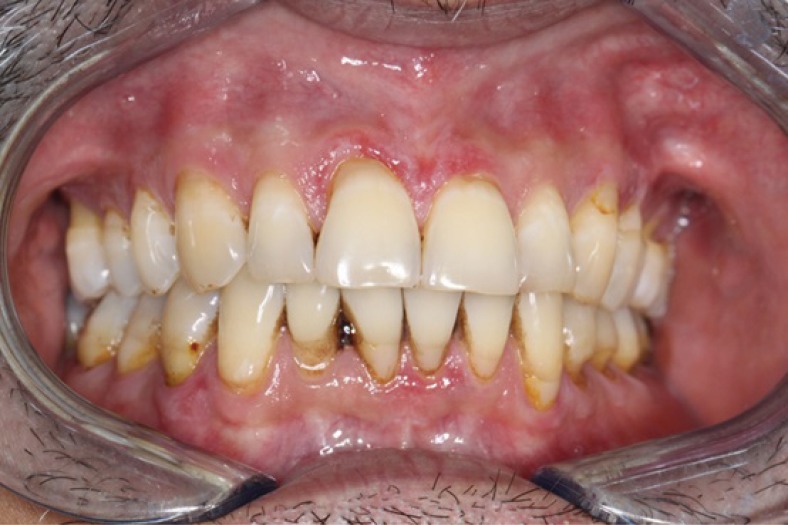
Reticular, erythematous lesions involving the maxillary and mandibular gingiva.

**Fig. (1b):**
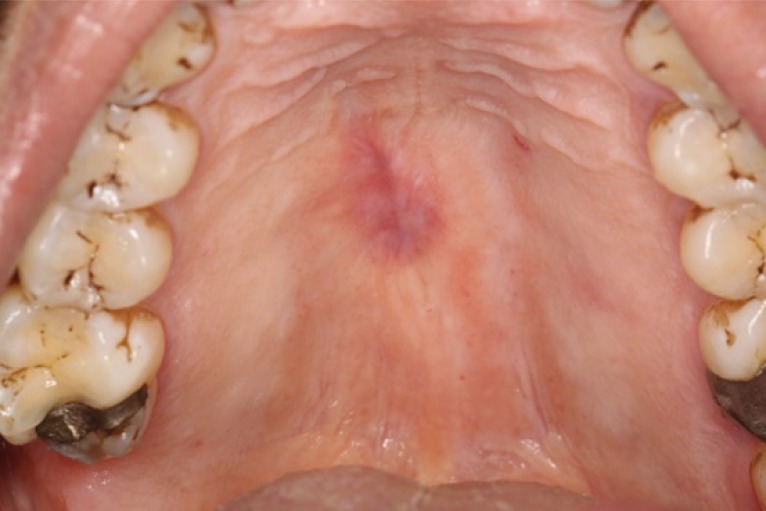
Post operative photograph of healed hard palate excisional biopsy.

**Fig. (2):**
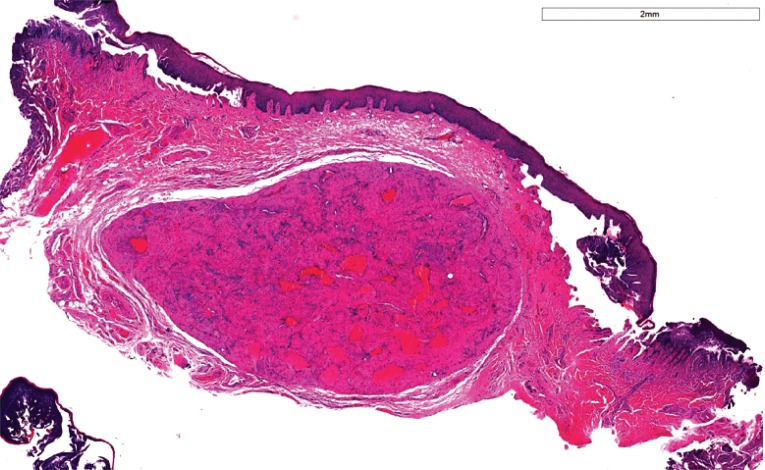
Mass of smooth muscle with small angulated vasculature. H and E, ×40.

**Fig. (3a):**
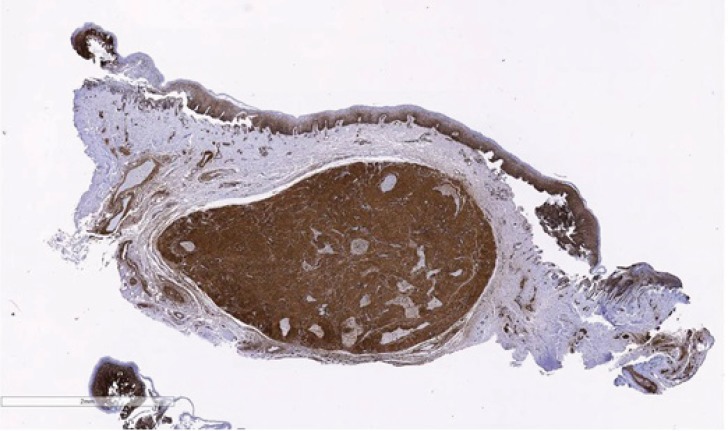
Lesional tissue showing positivity for smooth muscle actin.

**Fig. (3b):**
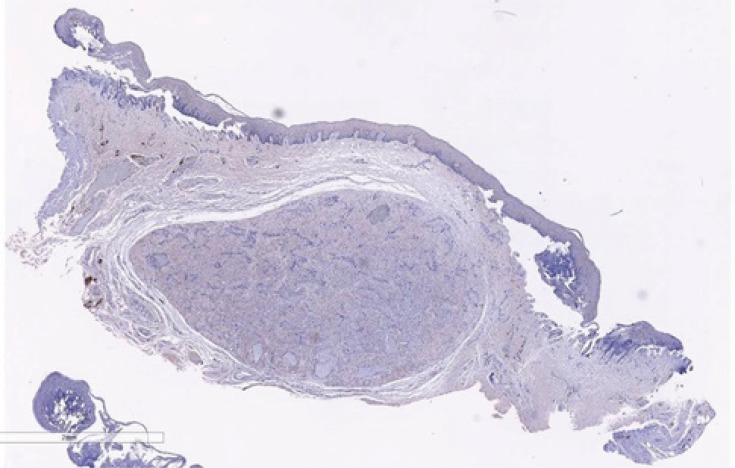
Lesional tissue showing negativity for S100 (IHC, ×40).

**Table 1:** Solid leiomyoma histopathological features [3].

Interlacing bundles of smooth muscle fibres, which may be interspersed by varying amounts of fibrous connective tissue
Bundles of fibers may form whorls because of their fasicular arrangement in varying planes
Nuclei are typically spindle shaped with blunt ends and quite vesicular
Mitotic figures are uncommon
Rare cells may exhibit nuclear pleomorphism
Necrosis and invasion are absent
Mucinous degeneration, hyalinization or fibrosis, and adipocytes can be seen, but these features are usually focal and more likely seen in larger lesions
Occasionally leiomyomas have a prominent fibroblastic or myxoid component.

**Table 2:** Differential diagnoses of an oral leiomyoma.

Fibroma
Neurofibroma
Lipoma
Leiomyosarcoma,
Myofibroma
Myopericytoma/haemangiopericytoma,
Schwannomas,
Salivary gland neoplasms
Vascular tumors such as lymphangioma and hemangioma
Soft tissue cysts such as dermoid cysts

**Table 3:** Other recent cases of oral leiomyoma.

Paper	Duration and Appearance	Site	Age and Gender	Diagnoses
Gonzalez Sanchez *et al* 2007 [11]	5mm diameter, red and painless mass of 4 months duration.	Hard palate, adjacent to upper left premolars.	57 year old male	Oral leiomyoma
Gaitan Cepeda *et al* 2008 (5 cases) [14]	Single, asymptomatic, slow growing nodule	Retromolar region, left submandibular region, lower lip and 2 involving the upper lip region	Avergage age over the 5 cases was 40.6 years old	Vascular leiomyoma
Nonaka *et al* 2010 [15]	Nodular growth	Tongue	38 year old male	Vascular leiomyoma
Gianluca *et al* 2011 [2]	8-year history of a well circumscribed, asymptomatic, brownish and smooth mass	Lower lip	49 year old male	Solid leiomyoma
Kaur and Gondal 2011 [7]	Greyish white and firm mass involving of one month history	Right submandibular region	32 year old male	Oral leiomyoma
Reddy *et al* 2011 [16]	Extra-oral swelling	Right body of the mandible	9 year old female	Leiomyoma
Alvarez *et al* 2012 [13]	Erythematous lesions of 4 months duration, about 1 cm in diameter	Tongue, cheek and floor of the mouth	8 month old female	Multiple leiomyomas
Montague *et al* 2013 (2 cases) [17]	Nodular growth of unspecified duration. Yellow-brown, soft nodule present for as long as patient could remember.	Anterior maxillary region Right mandibular vestibular area	51 year old female 18 year old female	Leiomyoma with extensive ossification
Veeresh *et al* 2013 [1]	Pale pink swelling of 2 months duration. Sessile and firm.	Palatal of right upper molar teeth	48 year old male	Angioleiomyoma

## References

[1] Veeresh M., Sudhakara M., Girish G., Naik C. (2013). Leiomyoma: A rare tumor in the head and neck and oral cavity: Report of 3 cases with review.. J. Oral Maxillofac. Pathol..

[2] Gianluca S., Marini R., Tonoli F., Cristalli M.P. (2011). Leiomyoma of oral cavity: Case report and literature review.. Ann. Stomatol. (Roma).

[3] Sharma A., Goyal G., Panwar S.S., Nangia R. (2015). Leiomyoma of oral cavity: A review.. Ann Dental Specialty.

[4] Baden E., Doyle J.L., Lederman D.A. (1994). Leiomyoma of the oral cavity: A light microscopic and immunohistochemical study with review of the literature from 1884 to 1992.. Eur. J. Cancer B Oral Oncol..

[5] Leung K.W., Wong D.Y., Li W.Y. (1990). Oral leiomyoma: case report.. J. Oral Maxillofac. Surg..

[6] Dutt K.C., Bindra S., Awana M., Talwar M., Lehl G. (2017). Intraosseous Leiomyoma of the Mandible: A case report of the rare entity and review of literature.. J. Maxillofac. Oral Surg..

[7] Kaur G., Gondal R. (2011). Oral leiomyoma.. J. Oral Maxillofac. Pathol..

[8] Glas E. (1905). Contribution to pathology of tongues.. Wien. Klin. Wochenschr..

[9] Luaces Rey R., Lorenzo Franco F., Gómez Oliveira G., Patiño Seijas B., Guitián D., López-Cedrún Cembranos J.L. (2007). Oral leiomyoma in retromolar trigone. A case report.. Med. Oral Patol. Oral Cir. Bucal.

[10] Fletcher C., Unni KK, Mertens F. (2002). WHO pathology and genetics of tumours of soft tissue and bone..

[11] González Sánchez M.A., Colorado Bonnin M., Berini Aytés L., Gay Escoda C. (2007). Leiomyoma of the hard palate: a case report.. Med. Oral Patol. Oral Cir. Bucal.

[12] Damm D.D., Neville B.W. (1979). Oral leiomyomas.. Oral Surg. Oral Med. Oral Pathol..

[13] Alvarez E, Laberry M, Ardilla C (2012). Multiple oral leiomyomas in an infant: A rare case, case reports in dentistry.

[14] Gaitan Cepeda L.A., Quezada Rivera D., Tenorio Rocha F., Leyva Huerta E.R., Mendez Sánchez E.R. (2008). Vascular leiomyoma of the oral cavity. Clinical, histopathological and immunohistochemical characteristics. Presentation of five cases and review of the literature.. Med. Oral Patol. Oral Cir. Bucal.

[15] Nonaka C.F., Pereira K.M., Miguel M.C. (2010). Oral vascular leiomyoma with extensive calcification areas.. Rev. Bras. Otorrinolaringol. (Engl. Ed.).

[16] Reddy B., Rani B.S., Anuradha Ch., Chandrasekhar P., Shamala R., Lingamaneni K. (2011). Leiomyoma of the mandible in a child.. J. Oral Maxillofac. Pathol..

[17] Montague L.J., Fitzpatrick S.G., Islam N.M., Cohen D.M., Bhattacharyya I. (2014). Extensively ossifying oral leiomyoma: A rare histologic finding.. Head Neck Pathol..

